# Morel–Lavallee lesions and number of surgeries for associated injuries predict surgical site infection risk following pelvic ring injury osteosynthesis

**DOI:** 10.1038/s41598-023-35488-8

**Published:** 2023-05-22

**Authors:** Chih-Yang Lai, Po-Ju Lai, I-Chuan Tseng, Chun-Yi Su, Yung-Heng Hsu, Ying-Chao Chou, Yi-Hsun Yu

**Affiliations:** 1 Department of Orthopedic Surgery, Linkou Branch, Musculoskeletal Research Center, Chang Gung Memorial Hospital, Chang Gung University, Taoyuan City, Taiwan; 2 Department of Orthopedic Surgery, Chang Gung Memorial Hospital, Taoyuan Branch, Taoyuan City, Taiwan; 3 Department of Orthopedic Surgery, Chang Gung Memorial Hospital, Keelung Branch, Keelung City, Taiwan

**Keywords:** Diseases, Medical research, Risk factors

## Abstract

We examined the incidence and causative factors of surgical site infection (SSI) following osteosynthesis for pelvic ring injury by reviewing the data of 97 consecutive patients with pelvic ring injuries treated between 2014 and 2019. Osteosyntheses, including internal or external skeletal fixation with plates or screws, were performed based on fracture type and patient’s condition. Fractures were treated surgically, with a 36 months minimum follow-up period. Eight (8.2%) patients experienced SSI. The most common causative pathogen was *Staphylococcus aureus*. Patients with SSI had significantly worse functional outcomes at 3, 6, 12, 24, and 36 months than those without. For patients with SSI, the average Merle d’Aubigné and Majeed scores at 3, 6, 12, 24, and 36 months after injury were 2.4, 4.1, 8.0, 11.0, and 11.3, and 25.5, 32.1, 47.9, 61.9, and 63.3, respectively. Patients with SSI had a higher likelihood of undergoing staged operations (50.0 vs. 13.5%, *p* = 0.02), more surgeries for associated injuries (6.3% vs. 2.5%, *p* = 0.04), higher likelihood of Morel–Lavallee lesions (50.0% vs. 5.6%, p = 0.002), higher incidence of diversional colostomy (37.5% vs. 9.0%, *p* = 0.05), and longer intensive care unit stay (11.1 vs. 3.9 days, *p* = 0.001) than those without. The contributing factors for SSI were Morel–Lavallee lesions (odds ratio [OR] 4.55, 95% confidence interval [95% CI] 3.34–50.0) and other surgeries for associated injuries (OR 2.37, 95% CI 1.07–5.28). Patients with SSI after osteosynthesis for pelvic ring injuries may have worse short-term functional outcomes.

## Introduction

Pelvic ring injuries are commonly observed following high-energy trauma. Patients with pelvic ring injuries have a relatively high mortality rate of 10–27%^1,2^. With improvements in in-hospital care, diagnostic modalities, resuscitation protocols, damage control procedures, osteosynthesis, evidence-based algorithms, and multidisciplinary teamwork, the morbidity and mortality rates have decreased over the past decade^3^. However, complications following major injuries remain high, owing to the complexity of fracture patterns, concomitant major organ injuries, and the requirement for multiple surgical interventions^4,5^.

Surgical site infection (SSI) is a complication of osteosynthesis that negatively impacts postoperative functional outcomes^6,7^. The incidence of SSI among orthopedic surgeries has been reported to be approximately 1–5%^8,9^. However, higher rates (2–6%) have been reported in cases of osteosynthesis for pelvic ring injury^7,10^. Some potential factors that might cause SSI after osteosynthesis for pelvic ring injury have been described, including Morel–Lavallee lesions, pelvic arterial embolization, obesity, open fractures, and injury severity^6,7,10^. However, limited evidence exists regarding postoperative functional outcomes for such patients. There are some functional outcome instruments for reporting outcomes after surgery for pelvic ring injury, including the Majeed score, Merle d’Aubigné score, Short-Form 36, and Iowa pelvic score^11^.

This study aimed to identify the incidence and causative factors of SSI following osteosynthesis for pelvic ring injury at a single trauma center. Additionally, the short-term functional outcomes of enrolled patients were reported and compared with those of patients without SSI. We hypothesized that patients with SSI would have worse functional performance than those without SSI.

## Methods

This retrospective chart review involving human participants was conducted in accordance with the ethical standards of the institutional and national research committee and with the 1964 Helsinki Declaration and its later amendments or comparable ethical standards. The study protocol was approved by the Institutional Review Board of Chang Gung Medical Foundation, Taoyuan City, Taiwan (IRB No.: 202000632B). Owing to the retrospective nature of the study, the need for informed consent was waived by the Institutional Review Board of Chang Gung Medical Foundation, Taoyuan City, Taiwan (IRB No.: 202200246B0).

We retrospectively reviewed the trauma registry data for all patients with pelvic ring injuries who underwent osteosynthesis at our center between January 2014 and January 2019. We included patients with pelvic ring injuries who underwent osteosynthesis and complete medical and functional performance follow-ups for at least 36 months. Those who underwent conservative treatments, had simultaneous acetabular fractures, were lost to follow-up, and missed or refused functional evaluations were excluded from the study.

### Resuscitative and operative strategies

All patients with pelvic ring injuries were resuscitated and managed according to the Advanced Trauma Life Support guidelines during triage at the emergency department^3^. Arterial embolization is a standard procedure during resuscitation for pelvic fractures at our institute. Arterial embolization was performed when patients did not respond to resuscitation and contrast extravasation was noted on computed tomography, after the exclusion of other sources of hemorrhage. Osteosynthesis was performed as early as possible when patient conditions were stable. The surgical approaches and fixation methods for definite treatments were primarily based on fracture patterns and concomitant visceral or skeletal injuries. Generally, open reduction and internal fixation (ORIF) surgery using ilioinguinal^12^ and Pfannenstiel approaches was the most common choice for ventral pelvic ring injuries. For patients whose fractures could be sufficiently reduced using closed techniques, closed reduction and internal fixation (CRIF) with percutaneous screw fixation were chosen. Conversely, CRIF with iliosacral screws or trans-iliac–trans-sacral screws (TITSs) was the first choice for osteosynthesis for dorsal pelvic ring injuries. However, ORIF with screws, plates, triangular osteosynthesis, or spinopelvic osteosynthesis was performed when anatomical reduction could not be achieved via a closed method, or in cases of both vertical and horizontal instabilities. Occasionally, external skeletal fixators were determined to be most suitable for osteosynthesis; otherwise, bridging treatments for stage operations were used because of concomitant visceral injuries of the abdomen.

### Applied classifications

Pelvic ring injuries were classified into the following three types based on the Arbeitsgemeinschaft für Osteosynthesefragen (AO) classification system (2018 revision): type A, stable pelvic ring injury; type B, partially unstable pelvic ring; and type C, completely unstable pelvic ring^13^. The Faringer and Jones–Powell classification systems were employed to identify the severity of open pelvic ring injuries and their connections with SSIs.

The Faringer classification was developed to classify open pelvic ring injury based on the anatomical location of the soft tissue injury as follows: Zone I (perineum, anterior pubis, medial buttock, and posterior sacrum), Zone II (medial thigh and groin crease), and Zone III (posterolateral buttock, iliac crest)^14^. The Jones–Powell classification was determined based on the combination of the anatomical region of the open wound and the mechanical stability of the pelvic ring and was classified as follows: Class 1 (stable pelvic ring), Class 2 (unstable pelvic ring without rectal or perineal wound), and Class 3 (unstable pelvic ring with rectal or perineal wounds)^15^.

### Definition of SSI

The patients’ demographic characteristics, injury severity score (ISS), new injury severity score (NISS), fracture patterns, functional outcomes, and microorganisms detected in the SSI were recorded. SSI was defined based on the criteria by the Centers for Disease Control and Prevention of the United States of America, clinical symptoms, and laboratory test results^16^.

The clinical symptoms of the SSI included local erythema and swelling of the surgical wound, purulent discharge, unexplained fever after the index surgery, poor wound healing, and the formation of a draining sinus from the surgical wound. Laboratory signs included leukocytosis with a left shift, elevated C-reactive protein levels, and positive wound culture results from a surgical wound sample. The initial management of patients with SSI included adequate surgical debridement and irrigation of the infected site. Empirical antibiotic therapy was initiated after tissue samples were collected at the time of debridement. The antibiotic regimen and duration of administration were modified according to the patients’ culture results and physical responses.

### Evaluations of functional outcomes

The functional outcomes were assessed based on the Merle d’Aubigné and Majeed scores at 3, 6, 12, 24, and 36 months after injury in the enrolled patients. The Merle d’Aubigné score was divided into three items: pain, mobility, and ability to walk, which ranged from 0 to 6 points (from worst to best condition)^17^ (SI Table 1). The Majeed score is a pelvic injury-specific functional assessment that includes seven items: pain, work, sitting, sexual intercourse, standing, unaided gait, and walking distance, with a score ranging from 0 to 100 points in order of decreasing disability^11,18^ (SI Table 2).

The current study data were analyzed using SPSS software (version 23.0; IBM Corp., Armonk, NY, USA). Continuous variables were compared using the two-tailed t-test and Mann–Whitney U test, while categorical variables were compared using the chi-square and Fisher’s exact tests. Multivariate logistic regression was used to determine the potential risk factors for SSI.

### Ethical approval

The study was conducted in accordance with the Declaration of Helsinki and was approved by the Institutional Review Board of Chang Gung Medical Foundation, Taoyuan City, Taiwan (IRB No.: 202000632B).

### Informed consent

Requirement for patient consent was waived due to the retrospective nature of the study.

## Results

Ninety-seven patients with pelvic ring injuries met the inclusion criteria during the study period. The demographic data of the enrolled patients are presented in Table 1. Overall, the incidence of SSI was 8.2% in this cohort. Among the chosen treatment approaches for ventral pelvic ring injury, ORIF was performed in 22 patients using the ilioinguinal approach and in 23 patients using the Pfannenstiel approach. Various approaches were applied to treat dorsal pelvic ring injuries, including 45 patients who underwent CRIF utilizing iliosacral screws or TITSs, seven who underwent ORIF for sacroiliac joint injuries, two who underwent triangular osteosynthesis, and 17 who underwent spinopelvic osteosynthesis.

**Table 1:** Demographic characteristics of the 97 patients who underwent osteosynthesis for pelvic ring injuries.

Age (years) mean (SD)	39.2 (17.2)
Sex, n (%)	
Male	41 (57.7%)
Female	56 (42.3%)
Diabetes, n (%)	5 (5.2%)
Smoker, n (%)	11 (11.3%)
BMI, mean (SD)	22.9 (3.6)
ISS, mean (SD)	18.9 (12.0)
NISS, mean (SD)	22.2 (12.0)
Open fractures, n (%)	19 (19.6%)
Faringer zone	
I	11 (57.9%)
II	1 (5.3%)
III	7 (36.8%)
Jones–Powell classification	
1	4 (21.1%)
2	4 (21.1%)
3	11 (57.8%)
AO/OTA classification^a^	
61-A	8 (8.2%)
61-B	43 (44.3%)
61-C	46 (47.4%)
Osteosynthesis	
ORIF	71 (73.1%)
CRIF or ESF	26 (26.9%)
Stage operation, n (%)	16 (16.5%)
Number of other surgeries, mean (SD)	2.9 (2.4)
Arterial embolization, n (%)	39 (40.2%)
Rectum injury, n (%)	6 (6.2%)
Urogenital injury, n (%)	12 (12.4%)
Morel–Lavallee lesions, n (%)	9 (9.3%)
Colostomy, n (%)	11 (11.3%)
SSI, n (%)	8 (8.2%)
ICU length of stay, days (SD)	4.5 (5.7)
Time to bony union, months (SD)	6.0 (1.8)
Follow-up, months (SD)	40.3 (12.7)

^a^The classification of pelvic fractures was based on the Arbeitsgemeinschaft für Osteosynthesefragen (AO/OTA) classification (2018 revision).

*BMI* body mass index, *CRIF* closed reduction and internal fixation, *ESF* external skeletal fixator, *ICU* intensive care unit, *ISS* injury severity score, *NISS* new injury severity score, *ORIF* open reduction and internal fixation, *SD* standard deviation, *SSI* surgical site infection.

Table 2 shows the demographic characteristics of the patients with SSI. The participants’ median age was 35 years (interquartile range: 28), the sex ratio was 1:3 (male: female), and the mean number of other surgeries for associated injuries was 6.3 (SD, 4.1). Three (37.5%) patients with open fractures were classified as having Faringer Zone I and Jones–Powell Class 3. Four (50.0%) patients presented with associated Morel–Lavallee lesions. All of them were first debrided at the time of pelvic osteosynthesis, and repeated debridement surgeries were performed where necessary. Four patients (50.0%) underwent staged operations for pelvic ring injuries, and five (62.5%) patients underwent arterial embolization to achieve hemostasis during resuscitation. All eight SSI patients were treated successfully with adequate surgical debridement (1–6 times) and antibiotic therapy for 2–8 weeks, and all fractures achieved union within 4–10 months. No chronic osteomyelitis or non-union occurred during follow-up. The most frequently observed causative pathogen of SSI was *Staphylococcus aureus*, which was isolated in four (50%) patients, with methicillin-resistant strains in three patients. Polymicrobial infections affected two (20%) patients in our study who presented with open pelvic ring injuries, and anaerobic organisms were found in both patients, including *Bacteroides* species.

**Table 2:** Demographic characteristics of the eight patients with surgical site infections.

Case	Sex	Age, years	ISS	NISS	Faringer zone/Jones–Powell classification (open fracture)	Morel–Lavallee lesion	Number of other surgeries*	Colostomy	AE	Number of debridement surgeries	Duration of antibiotic therapy, weeks	Time to union, months	Pathogens
1	Male	55	32	41	I/3	Yes	14	Yes	Yes	3	8	8	*E. coli, Bacteroides* species
2	Female	49	18	22	I/3	No	10	Yes	No	5	8	5	*Proteus*, MRSA, *B. fragilis*
3	Female	55	17	17	I/3	No	4	No	No	1	3	8	MRSA
4	Female	45	16	26	–	Yes	8	Yes	Yes	6	8	5	*E. coli*
5	Female	67	36	41	–	Yes	4	No	Yes	5	8	7	MRSA
6	Female	16	19	27	–	No	4	No	No	1	2	4	MSSA
7	Female	68	17	17	–	Yes	2	No	Yes	1	4	5	*E. coli*
8	Male	53	17	17	–	No	4	No	Yes	1	4	10	*Klebsiella pneumoniae*

*B. fragilis Bacteroides fragilis*, *E. coli Escherichia coli*, *ISS* injury severity score, *NISS* new injury severity score, *AE* arterial embolization, *MRSA* methicillin-resistant *Staphylococcus aureus*, *MSSA* methicillin-sensitive *Staphylococcus aureus*, *SSI* surgical site infection.

*The definition of “other surgeries” is surgeries for commitment injuries other than either osteosynthesis for pelvic ring injury or debridement surgery for surgical site infection of the pelvic ring.

We further compared the characteristics of patients with and without SSI (Table 3). We found that patients with SSI, compared to those without SSI, had a higher likelihood of undergoing stage operations (50.0% vs. 13.5%, *p* = 0.02), required more surgeries for associated injuries (6.3% vs. 2.5%, *p* = 0.04), had a higher likelihood of Morel–Lavallee lesions (50.0% vs. 5.6%, *p* = 0.002), had a higher incidence of diversional colostomy (37.5% vs. 9.0%, *p* = 0.05), and had longer intensive care unit (ICU) length of stay (11.1 vs. 3.9 days, *p* = 0.001). However, there were no significant between-group differences regarding ISS, NISS, fracture type severity, open or closed fractures, arterial embolization, or osteosynthesis methods. For the analysis of risk factors for SSI, multiple logistic regression analysis was used to identify potential factors, such as the presence of Morel–Lavallee lesions (odds ratio [OR] 4.55, 95% confidence interval [CI] 3.34–50.0) and the number of other surgeries for associated injuries (OR 2.37, 95% CI 1.07–5.28; Table 4).

**Table 3:** Comparison of patients with and without surgical site infections following osteosynthesis for pelvic ring injury.

	Patients with SSI (n = 8)	Patients without SSI (n = 89)	*p* value
Age, years, median	35 (IQR, 28)	38 (IQR, 31)	0.4
Sex, n (%)			0.5
Male	2 (25.0%)	39 (43.8%)	
Female	6 (75.0%)	50 (56.2%)	
Diabetes, n (%)	1 (12.5%)	4 (4.5%)	0.4
Smoker, n (%)	2 (25%)	9 (10.1%)	0.2
BMI, median	25.6 (IQR, 6.0)	21.9 (IQR, 5.5)	0.2
ISS, median	20 (IQR, 13–28)	16.5 (IQR, 8–24)	0.1
NISS, median	27 (IQR, 22–38)	17 (IQR, 12–29)	0.1
Open fracture, n (%)	3 (37.5%)	16 (18%)	0.2
AO classification^a^			0.06
61-A	0	8 (9.0%)	
61-B	1 (12.5%)	42 (47.2%)	
61-C	7 (87.5%)	39 (43.8%)	
Osteosynthesis			0.45
ORIF	5 (62.5%)	66 (74.2%)	
CRIF or ESF	3 (37.5%)	23 (25.8%)	
Stage operation, n (%)	4 (50.0%)r	12 (13.5%)	0.02*
Number of other surgeries, median	5 (IQR, 5)	2 (IQR, 2)	0.03*
Arterial embolization, n (%)	5 (62.5%)	34 (38.2%)	0.2
Rectum injury, n (%)	1 (12.5%)	5 (5.6%)	0.41
Urogenital injury, n (%)	2 (25%)	10(11.2%)	0.3
Morel–Lavallee lesions, n (%)	4 (50%)	5 (5.6%)	0.002*
Colostomy, n (%)	3 (37.5%)	8 (9.0%)	0.05
ICU length of stay, days, median	6 (IQR, 5.5)	3 (IQR, 5)	0.01*
Time to bony union, months, median	6 (IQR, 3.5)	6 (IQR, 1)	0.6
Follow up, months, median	36 (IQR, 3.5)	36 (IQR, 8.5)	0.5

*SSI* surgical site infection, *BMI* body mass index, *CRIF*, closed reduction and internal fixation, *ICU* intensive care unit, *ISS* injury severity score, *NISS* new injury severity score, *ORIF* open reduction and internal fixation, *IQR* interquartile range.

^a^The classification of pelvic fractures was based on the Arbeitsgemeinschaft für Osteosynthesefragen (AO/OTA) classification (2018 revision).

*Statistical significance.

**Table 4:** Results of multiple logistic regression analysis of risk factors for surgical site infections.

Risk factors	OR	Estimated 95% CI	*p* value
Morel–Lavallee lesion Number of other surgeries	4.55	3.34–50.0	0.004
	2.37	1.07–5.28	0.03

*SSI* surgical site infection, *OR* odds ratio, *CI* confidence interval.

Table 5 shows the comparisons of functional outcomes between patients with and without SSI. For patients with SSI, the average Merle d'Aubigné scores at 3, 6, 12, 24, and 36 months after injury were 2.4, 4.1, 8.0, 11.0, and 11.3 points, respectively, while the average Majeed scores for the same time points were 25.5, 32.1, 47.9, 61.9, and 63.3 points, respectively. We found that patients with SSI had worse functional outcomes (as represented by both Merle d’Aubigné and Majeed scores) at each time point, with significant differences from the outcomes of patients without SSI following osteosynthesis for pelvic ring injuries (all *p* < 0.05).

**Table 5:** Functional outcomes of patients with and without surgical site infections following osteosynthesis for pelvic ring injuries.

	Patients with SSI (n = 8)	Patients without SSI (n = 89)	*p* value
Merle d’Aubigné score (SD)			
3 months	2.4 (2.3)	5.7 (3.3)	0.006*
6 months	4.1 (2.7)	10.3 (3.8)	0.001*
12 months	8.0 (4.0)	14.1 (3.1)	0.003*
24 months	11.0 (2.8)	15.3 (2.7)	0.003*
36 months	11.3 (4.1)	15.6 (3.1)	0.02*
Majeed score (SD)			
3 months	25.5 (9.4)	38.0 (14.8)	0.006*
6 months	32.1 (12.4)	57.3 (16.6)	0.001*
12 months	47.9 (18.9)	72.9 (15.5)	0.007*
24 months	61.9 (12.4)	79.3 (13.3)	0.005*
36 months	63.3 (14.0)	81.6 (13.9)	0.007*

*SD* standard deviation, *SSI* surgical site infection.

*Statistical significance.

## Discussion

This study reviewed patients with SSI following osteosynthesis for pelvic ring injury and investigated the potential factors contributing to SSI. Moreover, short-term functional outcomes were evaluated for all enrolled patients to determine whether SSI worsened functional performance. As a result of this analysis, it can be concluded that Morel–Lavallee lesions and a higher number of concomitant surgeries may predispose to SSI. Furthermore, patients with SSI had worse functional outcomes at each evaluation time point.

Morel–Lavallee lesions are closed internal degloving injuries resulting from shear injury of the soft tissues surrounding the pelvis and are commonly associated with pelvic and acetabular fractures^19^. Morel–Lavallee lesions are often overlooked and undertreated, facilitating bacterial colonization and leading to subsequent infection of the surrounding soft tissues^20^. Steiner et al.^19^ in their study of 20 patients with Morel–Lavallee lesions with associated acetabular and pelvic ring injuries, advised open lesion debridement during or before osteosynthesis if the lesion was located at the site of the surgical approach. Tseng et al.^21^ stated that early percutaneous drainage with debridement, irrigation, and suction drainage to treat Morel–Lavallee lesions appeared safe and effective.

In our study, patients with SSI had a higher likelihood of Morel–Lavallee lesions than those without SSI (50.0% vs. 5.6%, *p* = 0.002). Meanwhile, the patients with Morel–Lavallee lesions were 4.55 times more likely to be infected than those without after osteosynthesis (OR 4.55, 95% CI 3.34–50.0). As a result, we identified Morel–Lavallee lesions as a potential SSI contributing factor. As part of our surgical strategy, adequate drainage of the accumulated hematoma by surgical debridement was performed once the lesion was diagnosed. Meanwhile, ORIF by an extended incision was avoided as much as possible because CRIF with percutaneous surgical procedures might be beneficial in reducing the incidence of SSI^21^.

The number of other surgeries for associated injuries was another risk factor for SSI in our study. However, no studies have investigated the relationship between multiple surgeries and SSI. Wise et al. developed a predictive score for determining SSI risk after orthopedic trauma surgery, suggesting that a higher injury severity score might be associated with a higher risk of SSI^22^. Li et al.^23^ reported that patients with SSI after acetabular fracture surgery were more likely to be obese, more severely injured, and have longer ICU stays. We hypothesized that the requirement of more surgeries would reflect the presence of more complications in the patient, implying reduced host resistance and increased risk of SSI. Hyperglycemia following trauma has been shown to be associated with SSI after orthopedic surgery^24^.

SSI is a major complication of osteosynthesis and may negatively impact postoperative functional outcomes^13^. For example, Naumann et al.^25^ reported that patients with SSI following operative fixation of closed ankle fractures had worse long-term functional outcomes than those without SSI. Henkelmann et al.^26^ reported that after fixation of tibial plateau fractures, patients with SSI had significantly poorer functional outcomes than those without SSI. However, no studies to date have described the functional outcomes of SSI with pelvic ring injuries following osteosynthesis. In our study, we found that patients with SSI had worse functional outcomes (represented by both the Merle d’Aubigné and Majeed scores) at each predetermined time point (3, 6, 12, 24, and 36 months after injury) than those without SSI following osteosynthesis for pelvic ring injuries. Despite the successful eradication of infections by a series of debridement surgeries and all the fractures achieving union, patients with SSI still had worse functional outcomes at each time-point evaluated.

Despite efforts to avoid study bias, our study has some limitations. First, the single-institution retrospective design was associated with risks of recall bias and a small sample size. The small sample size also restricted our ability to adequately identify independent risk factors for SSI. Some cases were excluded because of incomplete functional evaluations. In addition, functional performance evaluations were only reported 36 months postoperatively. A longer follow-up period might have altered the study results. However, we observed that functional evaluations plateaued at 24 and 36 months for most patients. Finally, only two scales (Merle d’Aubigné and Majeed scores) were adapted to evaluate functional outcomes. Different evaluation scales, such as the 36-item short-form survey and sexual function assessments, might be beneficial in evaluating various aspects of functional performance in these patients.

In conclusion, we identified that Morel–Lavallee lesions and the number of other surgeries for associated injuries were independent risk factors for SSI following osteosynthesis of pelvic ring injuries. Furthermore, patients with SSI had worse short-term functional outcomes after pelvic ring injuries. Based on these results, a protocol to identify potential or apparent Morel–Lavallee lesions and decrease the number of other surgeries might increase the functional performance of this cohort postoperatively.

## Supplementary Information

Supplementary Tables.

## Data Availability

All data generated or analyzed during this study are included in this published article.
