# Supplementary material for: Morel–Lavallee lesions and number of surgeries for associated injuries predict surgical site infection risk following pelvic ring injury osteosynthesis
Source: Sci Rep. 2023 May 22;13:8208. doi: 10.1038/s41598-023-35488-8 (PMC10203252; doi:10.1038/s41598-023-35488-8)
Supplement: Supplementary file 1 — Supplementary Tables. [file 41598_2023_35488_MOESM1_ESM.pdf]

SI Table 1

Merle d’Aubigné score

Criteria	*Assessment*	*Score*
Pain	Intense and permanent	0
	Severe scor even at night	1
	Severe when walking, prevents any activity	2
	Tolerable with limited activity	3
	Mild when walking, disappearing at rest	4
	Mild and inconsistent; normal activity	5
	No pain	6
Mobility	Ankylosis with bad position of hip	0
	No movement, pain or slight deformity	1
	Flexion <40°	2
	Flexion 40°-59°	3
	Flexion 60°-79°, foot can be reached	4
	Flexion 80°-90°, abduction of at least 15°	5
	Flexion exceeds 90°, abduction of 30°	6
Ability to walk	None	0
	Only with crutches	1
	Only with canes	2
	With one cane, less than an hour, very difficult without cane	3
	A long time with a cane, short time without cane and limping	4
	Without cane, slight limp	5
	Normal	6

SI Table 2

Majeed score

Criteria	*Assessment*	*Score*
Pain	Intense,continuous at rest	0-5
	Intense with activity	10
	Tolerable,but limits activity	15
	With moderate activity,abolished by rest	20
	Mild,intermittent,normal activity	25
	Slight,occasional or no pain	30
Work	No regular work	0-4
	Light work	8
	Change of job	12
	Same job, reduced performance	16
	Same job, same perormance	20
Sitting	Painful	0-4
	Painful if prolonged or awkward	6
	Uncomfortable	8
	Free	10
Sexual intercourse	Painful	0-1
	Painful if prolonged or awkward	2
	Uncomfortable	3
	Free	4
Standing	Bedridden or almost	0-2
	Wheelchair	4
	Two crutches	6
	Two sticks	8
	One stick	10
	No sticks	12
Gait unaided	Cannot walk or almost	0-2
	Shuffing small steps	4
	Gross limp	6
	Moderate limp	8
	Slight limp	10
	Normal	12
Walking distance	Bedridden or few metres	0-2
	Very limited time and distance	4
	Limited with sticks, difficult without prolonged standing	6
	One hour with a stick	8
	One hour without sticks slight pain or limp	10
	Normal for age and general condition	12
